# Functional analysis of *Agaricus bisporus* serine proteinase 1 reveals roles in utilization of humic rich substrates and adaptation to the leaf‐litter ecological niche

**DOI:** 10.1111/1462-2920.13350

**Published:** 2016-06-07

**Authors:** Mary N. Heneghan, Claire Burns, Ana M. S. B. Costa, Kerry S. Burton, Michael P. Challen, Andy M. Bailey, Gary D. Foster

**Affiliations:** ^1^ School of Biological SciencesLife Sciences Building, 24 Tyndall Avenue, University of BristolBristolBS8 1TQUK; ^2^ Department of Life SciencesInstitute of Technology, SligoAsh LaneSligoIreland; ^3^ Warwick HRI, University of WarwickWellesbourneWarwickCV35 9EFUK; ^4^ Present address: East Malling ResearchEast MallingKentME19 6BJUK; ^5^ Present address: Wellcome Trust Centre for Human Genetics, University of OxfordRoosevelt DriveOxfordOX3 7BNUK

## Abstract

*Agaricus bisporus* is a secondary decomposer fungus and an excellent model for the adaptation, persistence and growth of fungi in humic‐rich environments such as soils of temperate woodland and pastures. The *A. bisporus* serine proteinase SPR1 is induced by humic acids and is highly expressed during growth on compost. Three *Spr1* gene silencing cassettes were constructed around sense, antisense and non‐translatable‐stop strategies (pGRsensehph, pGRantihph and pGRstophph). Transformation of *A. bisporus* with these cassettes generated cultures showing a reduction in extracellular proteinase activity as demonstrated by the reduction, or abolition, of a clearing zone on plate‐based bioassays. These lines were then assessed by detailed enzyme assay, RT‐qPCR and fruiting. Serine proteinase activity in liquid cultures was reduced in 83% of transformants. RT‐qPCR showed reduced *Spr1* mRNA levels in all transformants analysed, and these correlated with reduced enzyme activity. When fruiting was induced, highly‐silenced transformant AS5 failed to colonize the compost, whilst for those that did colonize the compost, 60% gave a reduction in mushroom yield. Transcriptional, biochemical and developmental observations, demonstrate that SPR1 has an important role in nutrient acquisition in compost and that SPR1 is a key enzyme in the adaptation of *Agaricus* to the humic‐rich ecological niche formed during biomass degradation.

## Introduction

An ecosystem's carbon sequestration potential has gained increased interest due to current climate change concerns (Bailey *et al*., 2002; Strickland and Rousk, 2010). Forest ecosystems play a prominent role in global carbon cycling (Myneni *et al*., 2001), and understanding microbial involvement in decomposition in these ecosystems is required to estimate global carbon fluxes and their potential future changes (Zifcakova *et al*., 2011). Depolymerization of biopolymers is a key process in the cycling of carbon (Kahkonen and Hakulinen, 2011). With litter decomposition in temperate forests being mainly driven by fungal activity (Watling and Harper, 1998; Hattenschwiler *et al*., 2005; Steffen *et al*., 2007) these processes are likely to impact on the potential for carbon sequestration in such habitats. In terrestrial environments, basidiomycetes are probably the ecologically most significant group of fungi, involved in the breakdown and chemical conversion of litter components (Dix and Webster, 1995). They constitute a major fraction of the living biomass and are responsible for efficient degradation of many recalcitrant organic compounds in soil litter and the humic layer (Steffen *et al*., 2007). These humic substances are dark‐coloured products of biotransformation of plant material, chemically heterogeneous, stabilized physically by soil particle association and by micro‐aggregation into supramolecular associations (Piccolo, 2002; Six *et al*., 2002). Humic substances represent a significant component of stabilized carbon and nitrogen in soil and have important roles in global carbon and nitrogen cycling and in the regulation of the mobility and fate of plant nutrients and environmental contaminants (Murphy and Zachara, 1995; Weber, 1998; Christl *et al*., 2000; Zavarzina *et al*., 2004; Dong *et al*., 2006). Investigations into the mechanisms of humus biotransformation and the organisms responsible are particularly important under the changing environment and understanding of these processes may promote better predictions of the dynamics of soil organic matter.

Litter decomposing basidiomycetes are regarded as the key players in microbial lignin and humus degradation and produce a wide variety of oxidoreductases and hydrolytic enzymes capable of degrading all three principal litter components: cellulose, hemicellulose and lignin (Steffen *et al*., 2000, 2002; Martinez *et al*., 2005; Osono, 2007; Valaskova *et al*., 2007; Baldrian and Valaskova, 2008; Sinsabaugh, 2010; Snajdr *et al*., 2010; Burke *et al*., 2011). These extracellular enzymes can only be made if the fungus has an adequate nitrogen supply and so they are often accompanied by production of chitinases or proteases.

The basidiomycete *Agaricus bisporus* is a secondary decomposer fungus. Although it is not an effective competitor on un‐degraded plant debris, it grows well on partially‐decomposed plant material, hence is often abundant in the composted leaf and needle litter found in soils of temperate forests (Morin *et al*., 2012; Kerrigan *et al*., 2013). Given the wealth of information available concerning its commercial cultivation, coupled with the available genome data and transformation systems, *A. bisporus* is an ideal model for the study of fungal adaptation, persistence and growth in humic rich environments where nutrition is not readily available to primary degrading fungi (Burton *et al*., 1997a; Morin *et al*., 2012).

Serine proteinase activity has been identified as the major extracellular proteinase produced by *A. bisporus* mycelium growing in compost, in which nitrogen, a limiting factor of mushroom yield, is largely sequestered in the form of protein and microbial biomass, suggesting a nutritional role for this enzyme (Burton *et al*., 1997a). Extracellular mycelial serine proteinase is synthesized in response to humic‐associated protein rather than casein or glutamate as a nitrogen source (Burton *et al*., 1997a). High levels of serine proteinase activity are also produced in the stipe of *A. bisporus* mushrooms during post‐harvest senescence from which the SPR1 enzyme was purified and characterized biochemically (Burton *et al*., 1993, 1997b; Kingsnorth *et al*., 2001). Transgenic analysis of the *Spr1* promoter elements confirmed serine proteinase production in mycelium is regulated in response to specific nitrogen sources, and clearly demonstrated expression patterns that are appropriate for nutrient acquisition in mycelia and transport through the stipe for fruiting body production (Heneghan *et al*., 2009).

Recent genomic research has revealed complexity in the mechanisms of adaptation of *A. bisporus* to the humic‐rich ecological niche. A detailed analysis of genomic sequence coupled with expression data showed that twenty‐two genes specifically expressed in humic‐rich environments share a specific promoter motif; these include 3 serine proteinases, 3 laccases, 2 cutinases and 10 carbohydrate‐active genes (Morin *et al*., 2012). The expression ratios of these three humic‐responsive serine proteinases in humic:non‐humic environments were 235 for *Spr3*, 107 for *Spr4* and 103 for *Spr1* (Morin *et al*., 2012). Serine proteinase genes *Spr3* and *Spr4* (see Supporting Information Table S1) have 34.4% and 24.3% pairwise identity, respectively, to *Spr1*. Two further serine proteinases *Spr5* and *Spr6* have high transcript levels in compost but lower humic:non‐humic expression ratios. *Spr5* and *Spr6* have 42.4% and 31.1% pairwise identity to *Spr1*. Heneghan and colleagues (2009) also described *Spr2* and *Spr2*a, however although these have high pairwise identities to Spr1 (79.2% and 79.3%, respectively) and are within 20kb on the same scaffold, they are likely to play only minor roles in humic‐related nutrition as their transcript levels and humic:non‐humic expression ratios are much lower (Morin *et al*., 2012).

Whilst *A. bisporus* is well known for its commercial value as a cultivated mushroom, it is often overlooked that this and many other closely related species inhabit woodland and permanent pasture across most temperate environments, and thus there may be a vital role for proteases such as SPR1 in nutrient acquisition from humic associated material in soils and compost. Understanding the nutrient cycling role of fungi such as *A. bisporus* in ecosystems is a prerequisite to modelling and optimizing carbon management for sustainable forests. To fully elucidate the importance of the humic‐regulated serine proteinases in nutrient acquisition, a transgenic analysis of the role of *Spr1* was conducted. We describe here the silencing of the *Spr1* gene, the biochemical and molecular analysis of transformants and the monitoring of transformants through mushroom sporophore development.

## Results

Transgenic analysis of SPR1was performed using three different silencing constructs engineered to contain the *Spr1* cDNA in the sense (pGRsensehph) and anti‐sense (pGRantihph) orientations and as untranslatable‐sense (pGRstophph). Transformation of *A. bisporus* with these *Hph‐Spr1* silencing cassettes resulted in the generation of 8 sense (S), 14 antisense (AS) and 17 stop (ST) stable transformants. Wild type *A. bisporus* (WT) was also transformed with GFP as a transformation control. Transformants were maintained on MMP plates supplemented with hygromycin. Transformants displayed normal morphology in plate cultures when compared to wild type *A. bisporus*.

### Analysis of *A. bisporus Spr1*‐silenced transformants

*A. bisporus Spr1*‐silenced transformants, wild type and GFP control transformants were screened for proteinase activity using the plate‐based clearing zone assay. Average clearing zones for both non‐transformed control strain A15 and GFP transformants were similar: 3.16 mm for A15 and 3 mm for GFP transformants on SM medium with a gelatin overlay 17 day post‐inoculation (Supporting Information Fig. S2). Transformants exhibiting reduced or no proteinase activity were S1, S4, S5, AS1, AS3, AS5, AS10, AS13 ST4, ST6, ST8, ST9 and ST11. These were selected for further analysis along with three transformants exhibiting normal proteinase activity (S3, AS8, ST2).

Transformants were analysed by PCR and amplification using primers Hyg1 and Hyg2 resulted in the production of a 600 bp fragment in all *A. bisporus* transformants confirming transformation. The presence of an intact WT *Spr1* gene was confirmed in the wild‐type and all transformants using primers SprpromFwd and Spr‐z which bind in the *Spr1* promoter and gene to amplify a 1400 bp genomic DNA product showing that the *Spr1* locus had not been disrupted as a result of transformation. The presence of the intact silencing cassettes in pGRsensehph, pGRantihph and pGRstophph transformants, was also assessed by PCR, screening for presence of the 5′ and 3′ regions in separate reactions to avoid cross‐reaction with the genomic *Spr1* locus. Primers 004‐p1 and Spr‐z anneal within the *A. bisporus gpdII* promoter and *Spr1* cDNA of pGRsensehph and pGRstophph plasmids to give a 1075 bp 5′ product. Primers SPR‐x and 004‐p1 anneal within the *A. bisporus gpdII* promoter and *Spr1* cDNA of pGRantihph plasmid to yield a 1230 bp 5′ product. Primers Spr‐x and TrpCRev anneal within the *Spr1* cDNA and *A. nidulans TrpC* terminator of pGRsensehph and pGRstophph plasmids giving a 1263 bp 3′ product. Primers Spr‐z and TrpCRev anneal within the *Spr1* cDNA and *A. nidulans* TrpC terminator of pGRantihph plasmid resulting in a 1054 bp 3′ product. Amplification of these fragments confirmed the presence of the *Spr1* silencing cassette in transformants.

### Measurement of serine proteinase activity

**Figure 1:**
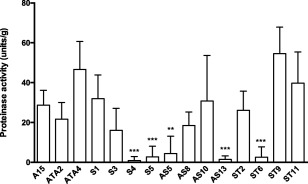
Normalized serine proteinase activity in 15 transformants growing in TM broth with humic fraction. A15 is the wild type strain, ATA2 and ATA4 are control transformants. Error bars are standard deviation of the mean of five biological replicates, with significance tested by Mann‐Whitney. *A. bisporus* transformants harbouring the *Spr1* sense (S), anti‐sense (AS) and untranslatable sense (ST) constructs.

Serine proteinase activity (normalized against dry weight) was assessed by inoculating sense‐transformants (S1, S3, S4, S5), antisense transformants (AS5, AS8, AS10, AS13) and sense‐stop transformants (ST2, ST6, ST9, ST11) along with wild type A15 and two transformed controls (ATA2, ATA4) into Treschow Media (TM) supplemented with humic acid (Fig. 1). Serine proteinase activity in control transformants (ATA2, ATA4) did not differ significantly from the non‐transformed control A15. The serine proteinase activities of five transformants, S4, S5, AS5, AS13 and ST6, were significantly reduced compared with the control strains (Fig. 1). The remaining transformants showed no significant difference to the control strains.

### Induction of serine proteinase by nitrogen source

**Figure 2:**
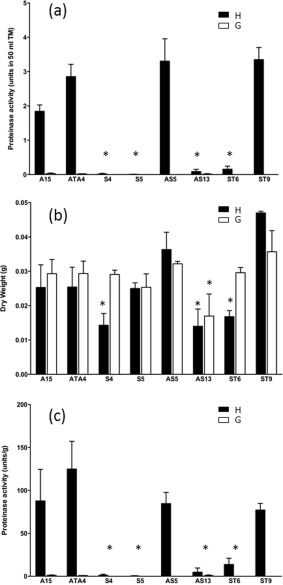
Induction of serine proteinase activity. A. Serine protease activity in nine transformants growing in TM broth with humic acid (H) or glutamate (G) as sole nitrogen source. B. Dry weights of transformants: A15 is the wild type strain, and ATA4 are control transformants. C. Normalized serine proteinase activity with respect to biomass. Error bars are standard error of the mean of five biological replicates. *A. bisporus* transformants harbouring the *Spr1* sense (S), anti‐sense (AS) and untranslatable sense (ST) constructs. Results were statistically analysed using *t*‐test and significant results are indicated by an asterisk (*).

To determine the effect of induction of *Spr1*, six transformants (S4, S5, AS5, AS13, ST6 and ST9), wild‐type A15 and transformed control ATA4 were grown in TM supplemented with humic fraction or glutamate to compare induction and non‐induction of serine proteinase activity respectively. Serine proteinase enzyme activity was not induced in the presence of glutamate as sole nitrogen source for any of the strains tested (Fig. 2A). For growth in humic fraction as sole nitrogen source, control transformant ATA4 did not differ significantly from the wild‐type A15, both showing high levels of SPR1 activity. Serine proteinase activity in four transformants (S4, S5, AS13 and ST6) was significantly down‐regulated compared to the controls A15 and ATA4 in humic fraction. No significant differences in serine proteinase activity were found between the remaining transformants and the controls.

The growth of the mycelial cultures was determined by measuring their dry weights at the end of the experiment (after 21 days growth). Control strains A15 and ATA4 and transformants S5, AS5 and AS13 had similar average dry weights for cultures grown on humic and glutamate as nitrogen source (Fig. 2B). However for AS13 both nutritional treatments resulted in lower dry weights than the controls. The dry weights of transformants S4 and ST6 were significantly reduced when grown on humic as sole nitrogen source while their growth on glutamate showed no difference compared with controls (Fig. 2B). Transformant ST9 had significantly increased dry weight when grown on humic fraction, compared with controls.

Normalized serine proteinase activity in media with either humic or glutamate nitrogen was significantly reduced in four transformants (S4, S5, AS13 and ST6) when compared with controls. In two transformants (AS5, ST9), there was no significant difference from non‐transformed and transformed controls (Fig. 2C).

### Quantification of *Spr1* transcripts

**Figure 3:**
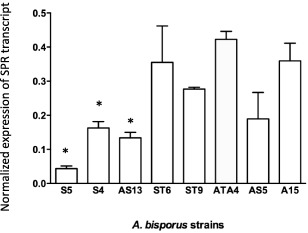
Quantification of serine proteinase transcripts determined by RT‐qPCR. Expression levels were normalized against 18S RNA. A15 is the wild type strain, ATA4 is a transformed control. Bars are means from SQRT scale. Error bars are +/− 0.5 standard error of the means of at least seven biological replicates *A. bisporus* transformants harbouring the *Spr1* sense (S), anti‐sense (AS) and untranslatable sense (ST) constructs and significance assessed using Unpaired *T*‐test *P* < 0.05 indicated by an asterisk.

Serine proteinase transcript levels were quantified in transformants S4, S5, AS5, AS13, ST6, ST9, transformed control ATA4 and wild‐type A15 (Fig. 3) using RT‐qPCR analysis. Transcript levels in the control transformant ATA4 did not differ significantly from the wild‐type, however three transformants (S4, S5 and AS13) displayed significantly reduced levels of *Spr1* transcripts.

**Figure 4:**
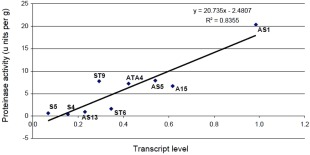
Linear regression analysis of the square root of the averages SPR1 transcript levels and proteinase activities for transformants S4, S5, AS1, AS5, AS13, ST6 and ST9 and controls (A15 and ATA4). The equation of the fitted line is: Serine proteinase activity = 20.7 x SPR1 transcript level–2.48.

### Correlation of serine proteinase activity and transcript levels

Serine proteinase transcript levels and enzyme activities of transformants (S4, S5, AS1, AS5, AS13, ST6 and ST9) and controls (A15 and ATA4) were compared. Linear regression analysis was performed on the square root of the averages of the *Spr1* transcript levels and proteinase activities (Fig. 4). A significant correlation was found between *Spr1* transcript levels and proteinase activities (correlation coefficient *R* ^2^ = 0.8355) (Fig. 4). The equation of the fitted line is: Serine proteinase activity = 20.7 x *Spr1* transcript level–2.48. This suggests that a unit change in transcript level (increase or decrease) results in a 20‐fold change in enzyme activity. Transformants S4, S5 and AS13, exhibiting low level of *Spr*1 transcript, also had very low serine proteinase enzyme activity (Fig. 4). Transformant AS1 showed increased levels of both serine proteinase activity and transcript abundance.

### Assessing the effect of *Spr1* silencing on *A. bisporus* growth on compost and fruiting

The impact of *Spr1* silencing on mycelial growth on compost and on mushroom production was determined for various selected transformants, non‐transformed control strain A15 and transformed control GFP1. Compost colonisation was assessed visually and subsequently, mushrooms were collected from 3 successive flushes and the weight of the mushrooms were recorded (Table 1). Transformant (AS5) failed to colonize the compost and hence produced no mushrooms. Four other transformants (S4, S5, ST4 and AS13) colonized the compost normally but gave significantly reduced yields of mushrooms (p<0.05) compared with the controls (non‐transformed A15 and transformed GFP1) (Table 1).

**Table 1:** Mushroom fruiting results for transgenic strains.

	Average weight of mushrooms harvested (g)			
Strain/Transformant	Flush 1	Flush 2	Flush 3	Total
A15 control	614	256	71	941
T‐ GFP control	534	188	24	746
S 1	509	159	83	751
S 3	502	299	133	934
S 4*	113	0	30	143
S 5*	137	50	55	242
AS 5*	0	0	0	0
AS 8	451	150	87	688
AS 10	377	61	72	510
AS 13*	147	0	29	176
ST 2	633	280	98	1012
ST 4*	205	145	30	381
ST 6	354	253	57	664
ST 9	648	226	6	880
ST 11	644	195	71	909

Transgenic strains were grown in three replicate pots and mushrooms harvested at maturity stage 2–3 (Hammond and Nichols, 1976) over a period of 30 days. Mushrooms were collected from three successive flushes and the total number of mushrooms and weight of mushrooms recorded. Yields of Sense (S), Antisense (AS) and Sense‐Stop (ST) transformants of *Spr1* were compared with two controls: non‐transformed A15 and GFP‐transformed T‐GFP. The LSD5% (Least Significant Difference) of the Total Yields was 288. The four transformants which were significantly different from the two controls are marked with an asterisk, *.

## Discussion

SPR1 has previously been demonstrated to be the major extracellular protease produced by *A. bisporus* during growth on humic fractions and this protease is also abundantly expressed during post‐harvest senescence of the fruiting body (Burton *et al*., 1993, 1994, 1997a, 1997b). To further investigate the importance of SPR1 in nutrient acquisition and adaptation of Agaricus to humic environments, a transgenic analysis of *Spr1* was conducted. A gene silencing approach was selected because it typically results in a diversity of transformants with varying silenced phenotypes, ranging from total inactivation of the gene to a small reduction in gene expression. Total inactivation, as achieved by gene disruption, may result in lethality to the fungus, whereas the suppression or downregulation mediated by gene silencing could permit a more practical functional analysis of critical genes (Heneghan *et al*., 2007; Kilaru *et al*., 2009). Furthermore, as *A. bisporus* is a multinucleate heterokaryon with two different nuclei, targeted gene knockouts of both alleles are likely to be difficult to achieve, whilst silencing should impact both as it is a dominant phenotype and should act similarly irrespective of the nuclear origin of the mRNA.

Three silencing strategies were employed using sense, antisense and sense‐stop constructs to trigger silencing of *Spr1*. Transformation of *A. bisporus* with the *Hph‐Spr1* silencing cassettes (pGRsensehph, pGRantihph, and pGRstophph) resulted in the generation of a number of stable hygromycin resistant colonies; 8 sense (S), 14 antisense (AS) and 17 stop (ST) transformants. Molecular analysis confirmed the integrity of the wild type *Spr1* gene in all transformants, demonstrating that gene knockouts had not occurred. The presence of a silencing cassette was confirmed in all transformants and given that previous work has established that only partial integration of the silencing cassette is necessary for silencing to occur (Namekawa *et al*., 2005; Heneghan *et al*., 2007) it was expected that amongst this population there would be some with a silencing phenotype. Of the 39 transformants obtained, 13 were impacted for protease production as indicated by reduction or abolition of clearing zones on plate‐based bioassay. Although these silencing frequencies were slightly lower than those reported for genes previously targeted in *A. bisporus* using complex hairpin‐based silencing constructs (Eastwood *et al*., 2008; Costa *et al*., 2009), it showed that these simpler cassettes were still effective in triggering silencing. The transformants with diminished clearing zones (S1, S4, S5, AS1, AS3, AS5, AS10, AS13, ST4, ST6, ST8, ST9 and ST11), indicative of a likely reduction in proteinase activity, along with three control transformants displaying normal proteinase activity, were selected for further analysis.

Whilst the plate‐based bioassay was indicative of reduced protease activity, it was not able to show which class of protease had been impacted. Therefore to specifically test for serine proteinase activity, a quantitative biochemical assay was employed using the serine protease‐specific substrate Succ‐Ala‐Ala‐Pro‐Phe‐pNA. This was conducted on 12 transformants (9 showing a reduction in proteinase activity and 3 demonstrating normal proteinase activity) grown in liquid culture supplemented with either humic or glutamic acid. Humic acid is known to induce SPR1 while nitrogen sources like glutamate do not induce expression (Burton *et al*., 1997a). Four of the transformants (S4, S5, AS13 and ST6) had significantly lower levels of serine proteinase activity than the controls when grown in humic fraction as sole nitrogen source. A strong correlation was found between *Spr1* transcript level and serine protease enzyme activity using regression analyses. Given the almost complete down‐regulation of extracellular serine proteinase activity in some of these transformants despite the presence of the inducing humic fraction, this suggests SPR1 would normally account for the majority of the protease activity during growth on purified humic fraction.

The failure to colonize compost in AS5 and the reduced fruiting of other transformants highlights the role of SPR1 during growth on complex humic rich substrates. The three plasmids used in this study expressed the silencing cassette using the constitutive glyceraldehyde‐3‐phosphate dehydrogenase (*gpdII*) promoter from *A. bisporus*. Previous work has demonstrated the efficacy of this regulatory unit in transgene expression in basidiomycetes (Costa *et al*., 2008, 2009). The use of this constitutive promoter caused silencing of SPR1 throughout the life cycle of the basidiomycete, but this clearly impacted on normal growth on compost and on mushroom production. We note that SPR has been implicated as a detrimental enzyme impacting on both the shelf‐life and nutritional quality of mushrooms post‐harvest and, therefore, any silencing‐based approach to reduce the impact of SPR1 post‐harvest, would need to be designed incorporating the use of an appropriately regulated promoter to avoid impacting on SPR1 during cultivation.

We are aware that Morin and colleagues (2012) highlighted some 40 proteinases that might potentially be involved in the degradation of compost proteins, but of these genes, only three (*Spr1*, *Spr3* and *Spr4*) were strongly up‐regulated during growth in mushroom compost, a complex humic‐rich substrate. Proteases *Spr3, Spr4, Spr5* and *Spr6* have 34%, 24%, 42.4% and 31.1% pair‐wise identities to *Spr1*, respectively, and this is below the degree of homology that would be required to facilitate cross‐silencing, so silencing of proteases other than SPR1 by our constructs is unlikely. Given that our experiments with purified humic fraction did not detect SPR3 or SPR4, it would suggest that they were not induced during growth in liquid culture on purified humic fraction. Their abundant transcript levels during growth on the more complex mushroom compost suggest that these other proteases are likely to be under a different regulatory pathway to SPR1. Even if other SPR proteases are expressed during growth on compost as inferred from the Morin and colleagues (2012) transcript data, the impact of silencing *Spr1* alone was sufficient to reduce the ability of *A. bisporus* to colonize compost, showing that SPR1 plays a crucial role in supporting growth on such substrates. In addition the reduced numbers and mass of the resulting mushrooms highlights the role of this protease in supporting the production of fruiting bodies and, therefore, any impairments in SPR1 production would impact on the fecundity of *A. bisporus*.

Fungi such as *A. bisporus* were comparatively common in woodland and permanent pasture, but are becoming less abundant. The clear role of extracellular proteases such as SPR1 in allowing this fungus to access nitrogen from complex sources has been highlighted here, however, these proteases are tightly regulated and are likely to be impacted by changes in land management. We would speculate that some of the reduced occurrences of such fungi may well correlate with altered land management practises such as the application of fertilizers or manures which will impact on the regulation of these proteases. Negative impacts of nitrogen application has been observed particularly for basidiomycete fungi in other studies (Egli, 2011; Paungfoo‐Lonhienne *et al*., 2015). Given the roles of such basidiomycetes in nutrient cycling, this is likely to alter the C‐ and N‐cycles in soils and hence alter the fertility or carbon sequestration capacities of such soils. What this means for the long‐term health of such soil is difficult to predict.

The results presented here clearly demonstrate the critical role serine proteinases have in *A. bisporus* adaptation to humic rich substrates and a functional analysis of the organism's adaptation to its ecological niche. The silenced transformants either failed to colonize the compost, or resulted in a reduction in mushroom yield and hence fecundity, when compared to controls. Although three serine proteinases (Spr1, Spr3 and Spr4) have been identified showing up regulation on compost, indicating a possible role in nutrient acquisition, it would appear that SPR3 and SPR4 cannot compensate for silencing *Spr1*, assuming no cross silencing has occured. Biochemical analysis of enzyme activity levels and molecular analysis of transcript levels provide further evidence that SPR1 has been significantly down‐regulated in these transformants suggesting an essential role for this serine proteinase in nutrient acquisition from humic associated material in compost. SPR1 clearly plays a major role in allowing *A. bisporus* to access nitrogen, and hence also carbon sources, during growth on humic‐rich substrates and is, therefore, likely to be a determining factor in N‐ and C‐ cycles in the humic‐rich ecological niche formed during degradation of plant material in the soil.

## Experimental procedures

### Strains and culture maintenance

*Escherichia coli* strain DH5α was the host strain for plasmid construction and maintenance. *Agrobacterium tumefaciens* strains AGL1 and LBA1126 (Bundock and Hooykaas, 1996) were used for *A. bisporus* transformations and cultured as previously described (de Groot *et al*., 1998). The *A. bisporus* commercial strain Sylvan A15 (Sylvan, Kittanning, PA 16201, USA) was used for transformations. Mycelia were routinely maintained at 25°C on malt‐peptone (Leach *et al*., 2004) agar plates and supplemented with 25 μg ml^−1^ hygromycin B to select for transformants.

### Construct design

Three different silencing constructs were designed and constructed to contain the *Spr1* cDNA in the sense, anti‐sense and sense orientation with a stop codon to prevent translation (untranslatable stop). *Spr1* cDNA was PCR amplified from pKing03 (contains *Spr1* cDNA cloned into pBK‐CMV) using primers engineered to contain the appropriate restriction sites (Supporting Information Table S2). *Spr1* cDNA in the sense orientation was amplified using primers Spr1‐p1 and Spr1‐p2 yielding a *Bsp*HI‐*Bam*HI fragment, primers Spr1‐p4 and Spr1‐p5 resulted in the amplification of *Spr1* cDNA in the antisense orientation as a *Afl*III‐*Bam*HI fragment and *Spr1* cDNA untranslatable stop was amplified using primers Spr1‐p3 and Spr1‐p2 yielding a *Bsp*HI‐*Bam*HI fragment. Fragments were cloned into the Agaricus Molecular Toolkit (Burns *et al*., 2006) generating plasmids p004sense, p004anti‐sense and p004stop, respectively, (Supporting Information Fig. S1A). These plasmids contained the *A. bisporus gpdII* promoter and *A. nidulans* terminator as previously described (Burns *et al*., 2006). To introduce restriction sites, these plasmids were then cloned into a polylinker plasmid (pSL1180, Invitrogen) generating the *Spr1* plasmids pSL004sense, pSL004anti‐sense and pSL004stop (Supporting Information Fig. S1B). The hygromycin cassette, *hph*, was isolated from phph004 (Burns *et al*., 2005) as a *Kpn*I‐*Sac*I fragment and cloned into pBluescript II (Stratagene). The *hph* cassette was then excised as a *Kpn*I‐*Bss*HII fragment and ligated to similarly digested pSL004Spr1 plasmids (pSL004sense, pSL004antisense and pSL004stop) thus linking the *hph* and *Spr1* cassettes to create plasmids pSLsensehph, pSLantihph, and pSLstophph (Supporting Information Fig. S1C). The *hph*‐*Spr1* cassettes were then excised as *Bgl*II‐*Spe*I fragments and ligated to pGREEN (Hellens *et al*., 2000) digested with *Bam*HI and *Spe*I to form binary plasmids pGRsensehph, pGRantihph and pGRstophph (Supporting Information Fig. S1D). Correct assembly of all constructs was confirmed by sequence analysis.

### Fungal transformations

*Agaricus bisporus* was transformed using *Agrobacterium tumefaciens* mediated transfection of gill tissue as previously described (Chen *et al*., 2000; Burns *et al*., 2005, 2006). Agaricus‐Agrobacterium co‐cultures were incubated at 20°C for 2–3 days before transferring the gill tissue to MMP agar containing 30 μg ml^−1^ hygromycin and 100 μg ml^−1^ cefotaxime. Selection plates were incubated at 25°C until transformed *A. bisporus* mycelia were visible (2–4 weeks) and transformants purified by three sequential subcultures under selection.

Genomic DNA was extracted from putative *A. bisporus* transformants and PCR screening was performed using Reddymix components (Abgene) with a general thermal cycling program of 95°C for 3 min, 30 cycles of (95°C for 30 sec, 50°C for 1 min, 72°C for 30 sec), and a final extension of 72°C for 10 min.

### Fruiting studies

To cultivate mushroom fruiting bodies transgenic strains were grown on autoclaved rye grain to produce grain spawn (Elliott, 1985) which was used to inoculate compost substrate prepared according to commercial practice (Eastwood *et al*., 2008). Each transgenic strain was grown in three replicate pots each containing 3 kg compost. Mushrooms were harvested at maturity stage 2–3 and weighed (Hammond and Nichols, 1976) over a period of 30 days. The yield data was statistically evaluated by analysis of variants using Genstat (17.1).

### Proteinase assays

Initial assessment of proteinase activity of wild type and transformed *A. bisporus* mycelia was screened by a clearing zone method. *A. bisporus* mycelia were inoculated onto SM medium with an overlay of 0.4% gelatin (w/v) in SM medium and examined after 17 days followed by staining with 0.1% (w/v) amido black in 7% (w/v) acetic acid and destained overnight using 7% acetic acid.

Expression of serine proteinase activity in liquid culture was determined by culturing *A. bisporus* mycelia of wild type, transformants, and transformed control in Treschow medium (TM; Treschow, 1944) supplemented with either humic fraction (0.94 g/l) or glutamate (5mM) and incubated at 25°C for 21 days. Dry weight was assessed and proteinase activity was measured spectrophotometrically using 0.15 mM Suc‐Ala‐Ala‐Pro‐Phe‐pNA substrate in 0.1 M sodium phosphate buffer (pH 7.0) with the OD measured continuously at 405 nm (Burton *et al*., 1997a). Five biological replicate cultures of each transformant or control were prepared. The protease activity per 50 ml of medium was determined and normalized to dry weight. One unit of proteinase activity was defined as an increase of one absorbance per minute.

Data of mycelial serine proteinase activities were subjected to square root (SQRT) transformations to stabilize the variance prior to analysis of variance (ANOVA). To analyse the correlation between serine proteinase enzyme activity and transcript levels, linear regression analysis was conducted on the square root of the averages of *Spr1* transcripts and proteinase activities. Mushroom weight yield data were statistically analysed by ANOVA.

### RT‐qPCR analysis

Total RNA was extracted from freeze‐dried mycelia using the method described by Sreenivasaprasad (2000). The RNA pellets were dried and dissolved in 100 μl of DMPC‐treated water. RNA was treated with DNase (Promega) prior to storage and preparations were confirmed free of contaminating genomic DNA by PCR amplification with primers SDH_Ab_F and SDH_Ab_R that span an intron of *SDH* gene of *A. bisporus* (Costa *et al*., 2009). Three independent RNA extractions were performed for each strain tested. cDNA synthesis was performed using 1 μg total RNA, with random hexamer primers and Thermoscript RT‐PCR System (Invitrogen).

The *SPR 1* transcripts were assessed using Q‐PCR MasterMix Plus for SYBR Green I w/o UNG (Eurogentec) on the ABI PRISM 7900HT Sequence Detection System (Applied Biosystems) using primers qSPR1_F and qSPR1_R that amplify a 62 bp region from *SPR1* cDNA. Expression levels of target genes were normalized against *A. bisporus* 18S rRNA using primers q18S_Ab_F and q18S_Ab_R, as described in Eastwood and colleagues (2008).

Two microliters of diluted cDNA products (1:3) was used in 15‐μL RT‐qPCR reactions with 500 nM of each primer and 2 μl Reaction buffer. Thermocycling parameters were: 50°C for 2 min; 95°C for 10 min; 40 cycles of 95°C for 15 s, 60°C for 1 min. Three replicate samples were run in 384‐well plates and sealed with optically clear heat seal film (Applied Biosystems). Standard curves were made using four serial dilutions (1, 1/4, 1/16, 1/64) of cDNA from the non‐silenced host. Negative controls (water, the non‐silenced host and a transformed control *A. bisporus*) were included in each experiment.

## Supporting information

Additional supporting information may be found in the online version of this article at the publisher's web‐site:

**Fig. S1.** Construction of *Spr1* silencing cassettes.
A. *Spr1* cDNA was cloned into the Basidiomycete Molecular Toolkit (Burns *et al*., 2006) generating plasmids p004sense, p004stop and p004 anti‐sense.
B. To introduce restriction sites, these plasmids were then cloned into a polylinker plasmid (pSL1180, Invitrogen) generating the *Spr1* plasmids pSL004sense, pSL004anti‐sense and pSL004stop.
C. The hygromycin cassette, *hph*, was isolated from phph004 (Burns *et al*., 2005) and cloned into pBluescript II (Stratagene). The *hph* cassette was then excised as a *Kpn*I‐*Bss*HII fragment and ligated to similarly digested pSL004Spr1 plasmids thus linking the *hph* and *Spr1* cassettes to create plasmids pSLsensehph, pSLstophph, and pSLantihph.
D. The *hph*‐*Spr1* cassettes were then excised as *Bgl*II‐*Spe*I fragments and ligated to pGREEN to form binary plasmids pGRsensehph, pGRstophph and pGRantihph.

**Fig. S2.** Proteinase clearing zone assays on milk or gelatin‐amended agar of non‐transformed control strain A15 (WT), GFP transformed *Agaricus bisporus* and hygromycin resistant sense (S), antisense (AS) and stop (ST) transformants. Colony and clearing zone diameters were measured 17 days after inoculation. (A) Control wild‐type A15 and GFP transformed *Agaricus bisporus*. (B) *Spr1*‐sense transformants. (C) *Spr1*‐antisense transformants. (D) *Spr1*‐stop transformants.

**Table S1.** Serine protease protein IDs and accession numbers.

**Table S2.** Sequence of primers used in the construction of molecular constructs, PCR analysis of transformants and other analysis. Incorporated restriction enzyme sites are underlined.
