# Supplementary material for: Functional analysis of Agaricus bisporus serine proteinase 1 reveals roles in utilization of humic rich substrates and adaptation to the leaf‐litter ecological niche
Source: Environ Microbiol. 2016 Jun 7;18(12):4687–96. doi: 10.1111/1462-2920.13350 (PMC5215592; doi:10.1111/1462-2920.13350)
Supplement: Supplementary file 1 — Fig. S1. Construction of Spr1 silencing cassettes. A. Spr1 cDNA was cloned into the Basidiomycete Molecular Toolkit (Burns et al., 2006) generating plasmids p004sense, p004stop and p004 anti‐sense. B. To introduce restriction sites, these plasmids were then cloned into a polylinker plasmid (pSL1180, Invitrogen) generating the Spr1 plasmids pSL004sense, pSL004anti‐sense and pSL004stop. C. The hygromycin cassette, hph, was isolated from phph004 (Burns et al., 2005) and cloned into pBluescript II (Stratagene). The hph cassette was then excised as a KpnI‐BssHII fragment and ligated to similarly digested pSL004Spr1 plasmids thus linking the hph and Spr1 cassettes to create plasmids pSLsensehph, pSLstophph, and pSLantihph. D. The hph‐Spr1 cassettes were then excised as BglII‐SpeI fragments and ligated to pGREEN to form binary plasmids pGRsensehph, pGRstophph and pGRantihph. [file EMI-18-4687-s001.docx]

Fig. S1.

✂

✂

✂

*Sac*I *Sac*II

*Kpn*I

Spr1

pBluescript

A

*A. bisporus gpdII* promoter

terminator

B

*Kpn*I

*Bss*HII

*Sac*I *Sac*II

Spr1

pSL1180

*A. bisporus gpdII* promoter

terminator

✂

✂

C

D

*hph* cassette

*Spr1* cassette

*Spe*I

LB

RB

*Bgl*II

pGREEN

*Kpn*I

*hph* cassette

*Spr1* cassette

*Bss*HII *Bgl*II

pSL1180

*Spe*I

✂

✂

✂

pSL1180

terminator

Spr1

*Kpn*I

*A. bisporus gpdII* promoter

*Sac*I *Sac*II

*Bss*HII

✂

✂

*Bss*HII

*Bss*HII

*hph*

*A. bisporus gpdII* promoter

*Kpn*I

terminator

pBluescript

*Sac*I

*hph*

*A. bisporus gpdII* promoter

*Kpn*I

terminator

pBluescript

*Sac*I
