# Supplementary material for: Functional analysis of Agaricus bisporus serine proteinase 1 reveals roles in utilization of humic rich substrates and adaptation to the leaf‐litter ecological niche
Source: Environ Microbiol. 2016 Jun 7;18(12):4687–96. doi: 10.1111/1462-2920.13350 (PMC5215592; doi:10.1111/1462-2920.13350)
Supplement: Supplementary file 2 — Fig. S2. Proteinase clearing zone assays on milk or gelatin‐amended agar of non‐transformed control strain A15 (WT), GFP transformed Agaricus bisporus and hygromycin resistant sense (S), antisense (AS) and stop (ST) transformants. Colony and clearing zone diameters were measured 17 days after inoculation. (A) Control wild‐type A15 and GFP transformed Agaricus bisporus. (B) Spr1‐sense transformants. (C) Spr1‐antisense transformants. (D) Spr1‐stop transformants. [file EMI-18-4687-s002.pptx]

## Slide 1
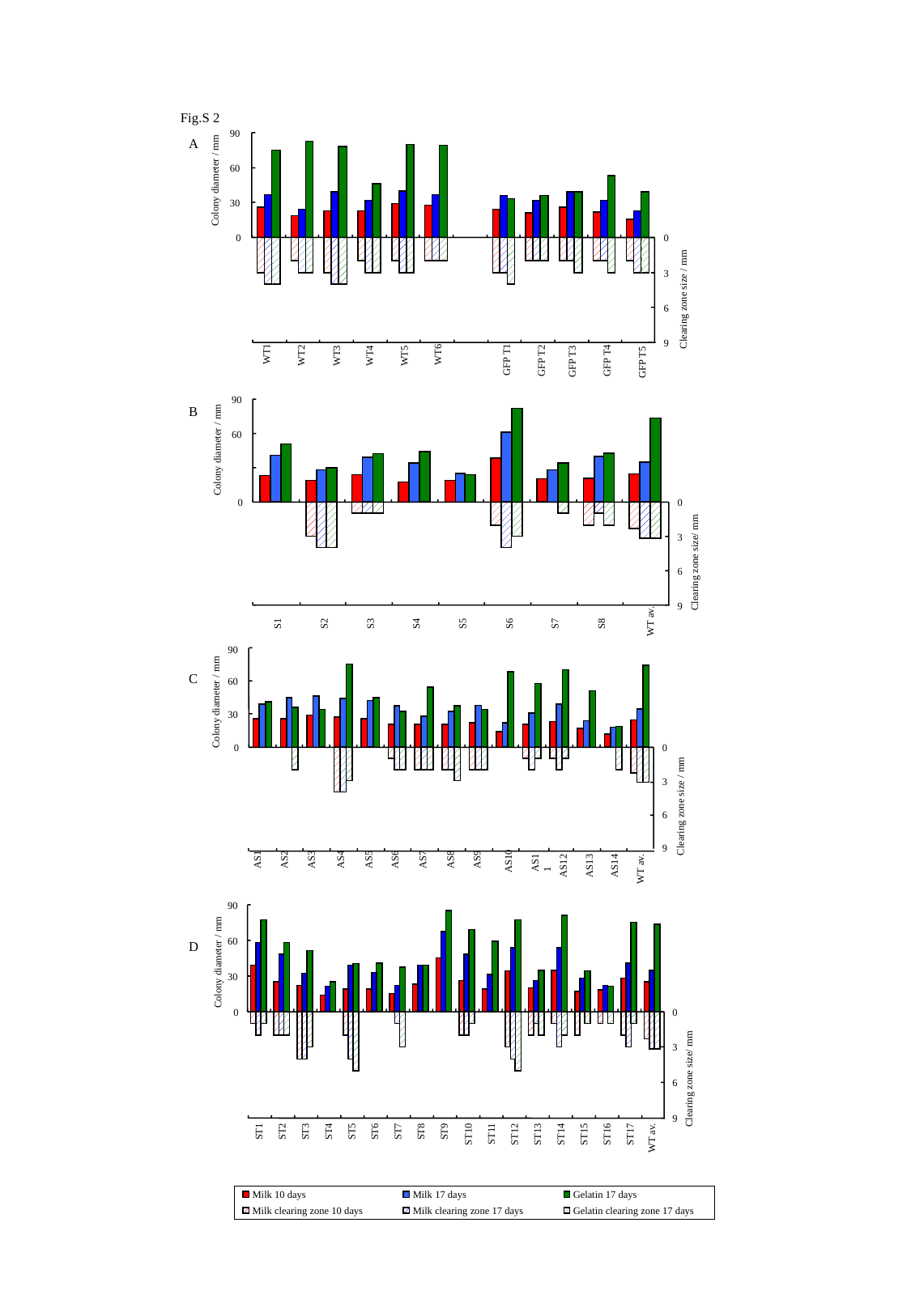

Fig.S 2
90
60
Colony diameter / mm
30
0
0
3
Clearing zone size / mm
6
9
WT5
WT2
GFP T5
WT4
WT3
GFP T1
GFP T3
WT6
GFP T4
WT1
GFP T2
A
90
B
60
Colony diameter / mm
0
0
Clearing zone size/ mm
3
6
9
S1
S2
S3
S4
S5
S6
S7
S8
WT av.
90
60
Colony diameter / mm
30
0
0
Clearing zone size / mm
3
6
9
AS10
AS1
AS2
AS3
AS4
AS5
AS6
AS7
AS8
AS9
AS13
AS11
WT av.
AS14
AS12
C
90
60
Colony diameter / mm
30
0
0
3
Clearing zone size/ mm
6
9
ST1
ST2
ST3
ST4
ST5
ST6
ST7
ST8
ST9
ST10
ST11
ST12
ST13
ST14
ST15
ST16
ST17
WT av.
D
Milk 10 days
Milk 17 days
Gelatin 17 days
Milk clearing zone 10 days
Milk clearing zone 17 days
Gelatin clearing zone 17 days
