# Supplementary material for: Functional analysis of Agaricus bisporus serine proteinase 1 reveals roles in utilization of humic rich substrates and adaptation to the leaf‐litter ecological niche
Source: Environ Microbiol. 2016 Jun 7;18(12):4687–96. doi: 10.1111/1462-2920.13350 (PMC5215592; doi:10.1111/1462-2920.13350)
Supplement: Supplementary file 3 — Table S1. Serine protease protein IDs and accession numbers. [file EMI-18-4687-s003.docx]

**Table S1** Serine protease protein IDs and accession numbers

*Spr1* (Protein ID194648; Accession XM_006463989)

*Spr2* (Protein ID 120844; Accession XM_006463984)

*Spr2*a (Protein ID 120846; Accession XM_006463987),

*Spr3* (Protein ID212650; Accession XM_00646079),

*Spr4* (Protein ID68978; Accession XM_006457256.1)

*Spr5* (Protein ID 133541; Accession XM_006454865)

*Spr6* (Protein ID 183321; Accession XM_006458174)
