# Supplementary material for: Functional analysis of Agaricus bisporus serine proteinase 1 reveals roles in utilization of humic rich substrates and adaptation to the leaf‐litter ecological niche
Source: Environ Microbiol. 2016 Jun 7;18(12):4687–96. doi: 10.1111/1462-2920.13350 (PMC5215592; doi:10.1111/1462-2920.13350)
Supplement: Supplementary file 4 — Table S2. Sequence of primers used in the construction of molecular constructs, PCR analysis of transformants and other analysis. Incorporated restriction enzyme sites are underlined. [file EMI-18-4687-s004.docx]

Table S2. Primers used in this study with relevant restriction sites underlined.

| Primer name | Sequence (5'-3') |
| --- | --- |
| Spr1-p1 | CCCGTCATGATGCATTTCTCTTTGTCT |
| Spr1-p2 | CCCGGATCCGCAAAGTGTATATTCCTT |
| Spr1-p3 | CCCGTCATGATGCATTTCTCTTAGTCT |
| Spr1-p4 | CCCGGATCCACCGACGATGCATTTCTC |
| Spr1-p5 | CCCACATGTGCAAAGTGTATATTCCTTGAC |
| qSPR1_F | GTTCTTGGATCGGTAGCAACACT |
| qSPR1_R | GGGAGTTGCCATGGAAGTTC |
| q18S_Ab_F | TCGCCGCTCCCTTGGT |
| q18S_Ab_R | GCATCGCCGGCACAA |
| SDH_Ab_F | TCACGTAAGAGACGCGAACA |
| SDH_Ab_R | AACGCAACTCGTGGTACTCA |
| Hyg1 | GCGTGGATATGTCCTGCGGG |
| Hyg2 | CCATACAAGCCAACCACGG |
| 004-p1 | CCCGCGTCTCGAATGTTCTC |
| Spr-z | AGAATGAAGCACGAGCGTCG |
| SPR-x | GCCAACTTCAAGGCCAAGGT |
| TrpCRev | GCACTCTTTGCTGCTTGGAC |
| SprpromFwd | CCGCGCAACATATGTATGTGAGAG |
